# Epizootic Hemorrhagic Disease Virus Serotype 8, Italy, 2022

**DOI:** 10.3201/eid2905.221773

**Published:** 2023-05

**Authors:** Alessio Lorusso, Stefano Cappai, Federica Loi, Luigia Pinna, Angelo Ruiu, Giantonella Puggioni, Annalisa Guercio, Giuseppa Purpari, Domenico Vicari, Soufien Sghaier, Stephan Zientara, Massimo Spedicato, Salah Hammami, Thameur Ben Hassine, Ottavio Portanti, Emmanuel Breard, Corinne Sailleu, Massimo Ancora, Daria Di Sabatino, Daniela Morelli, Paolo Calistri, Giovanni Savini

**Affiliations:** Istituto Zooprofilattico Sperimentale dell’Abruzzo e del Molise “Giuseppe Caporale”, Teramo, Italy (A. Lorusso, M. Spedicato, O. Portanti, M. Ancora, D. Di Sabatino, D. Morelli, P. Calistri, G. Savini);; Istituto Zooprofilattico Sperimentale della Sardegna, Sassari, Italy (S. Cappai, F. Loi, L. Pinna, A. Ruiu, G. Puggioni);; Istituto Zooprofilattico Sperimentale della Sicilia, Palermo, Italy (A. Guercio, G. Purpari, D. Vicari);; Institut de la Recherche Vétérinaire de Tunisie, Tunis, Tunisia (S. Sghaier);; Laboratoire de Santé Animale, Maisons–Alfort, France (S. Zientara, E. Breard, C. Sailleu);; Universitè de la Manouba, Tunisia (S. Hammami);; Commissariat Régional au Développement Agricole de Nabeul, Nabeul, Tunisia (T. Ben Hassine)

**Keywords:** epizootic hemorrhagic disease virus, EHDV, serotype 8, bluetongue, viruses, vector-borne infections, Italy, Southern Europe, Tunisia

## Abstract

We describe the detection of epizootic hemorrhagic disease virus (EHDV) serotype 8 in cattle farms in Sardinia and Sicily in October–November 2022. The virus has a direct origin in North Africa; its genome is identical (>99.9% nucleotide sequence identity) to EHDV serotype 8 strains detected in Tunisia in 2021.

The World Organisation for Animal Health (WOAH) lists epizootic hemorrhagic disease (EHD) as a disease of wild and domestic ruminants caused by EHD virus (EHDV). EHDV is related to bluetongue virus (BTV), the etiologic agent of bluetongue, a disease of ruminants. Both viruses belong to the genus *Orbivirus* and circulate in multiple serotypes (*1*,*2*). Their viral genomes consist of 10 segments (S1–S10) of double-strand RNA; the structural outer capsid protein (coded by S2) determines serotype specificity. Both viruses cause similar clinical signs in cattle and are transmitted by *Culicoides* spp. biting midges. Bluetongue primarily affects sheep and in recent decades has been described multiple times in the European Union (EU), causing devastating repercussions on animal trade (*3*). Most bluetongue outbreaks in Europe had a direct origin in North Africa because of wind-driven dissemination of BTV-infected midges from this region (*1*,*4*–*7*). We describe detection of EHDV serotype 8 (EHDV-8) in cattle in Italy, as a follow-up to our previous studies on EHDV-8 (GenBank accession nos. OP381190–9) in Tunisia in 2021 (*8*).

On October 25, 2022, respiratory distress, erosions of the muzzle and oral mucosa, and drooling were reported in 3 cattle at farm 1, located near the city of Trapani, Sicily (Figure); serum and whole blood samples were collected. On October 28, 2022, clinical signs consisting of inappetence, cyanosis and edema of the tongue, conjunctivitis, and fever were reported in an animal at farm 2, located in Arbus Municipality of Sardinia (Figure). On November 3, that animal died; spleen was collected at necropsy, along with whole blood from 3 additional symptomatic cattle on the farm. On November 4, at farm 3 (in Arbus Municipality) and farm 4 (in nearby Guspini Municipality), 3 cattle showed similar signs, and whole blood and serum samples were collected (Figure).

**Figure:**
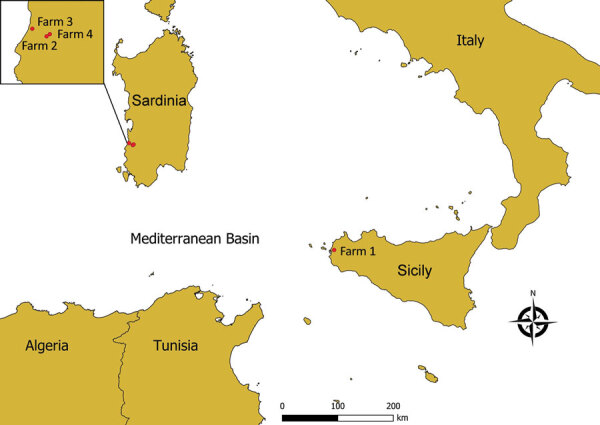
Geographic locations for detection of epizootic hemorrhagic disease virus serotype 8, Italy, 2022. Red dots indicate locations of the 4 farms involved. Inset map details locations of the 3 farms affected in the Arbus Municipality of Sardinia.

We tested 1 EDTA blood sample per animal and spleen samples for EHDV RNA by using a VetMAX EHDV Kit (Thermo Fisher Scientific, https://www.thermofisher.com). We developed a real-time reverse transcription PCR (rRT-PCR) specific for the S2 of the EHDV-8 strain detected in Tunisia in 2021 (EHDV-8 TUN 2021) because the available test designed for the S2 segment of the EHDV-8 reference serotype (isolated in Australia in 1982) did not detect EHDV-8 TUN 2021 (*8*). Primer nucleotide sequences were EHDV_Ser8varNEW_fwd AGAGATGAAGATCGCGAGGA and EHDV_Ser8varNEW_rev GAATCACACGCGCTCACTAA; the probe nucleotide sequence was EHDV_Ser8varNEW_Probe FAM-ACGGATGAGATACGGAACATACGGGG-TAMRA. We prepared the master mix by using TaqMan Fast Virus 1-Step (Thermo Fisher Scientific) and a final concentration of 400 nmol (primers) and 200 nmol (probe). After we performed RNA denaturation at 95°C for 3 min, we added 5 μL of RNA to 20 µL of mix and achieved amplification as follows: 45°C for 10 min, 95°C for 10 min, then 40 cycles of 95°C for 15 s, and finally 60°C for 1 min. We performed whole-genome sequencing (WGS) (*9*) on selected samples. We tested serum samples collected from all animals with a competitive ELISA, ID Screen EHDV Competition (Innovative Diagnostics, https://www.innovative-diagnostics.com), and by virus neutralization (*10*). We attempted virus isolation by using rRT-PCR–positive blood samples on Vero cells (*8*).

All sampled animals from Sardinia and Sicily were positive for EHDV RNA (cycle threshold 23–28). Genotyping confirmed the presence of EHDV-8 TUN 2021–like strains. We reported the outbreak to Italy’s Ministry of Health, which notified WOAH and the European Commission, which imposed animal movement restrictions within a 150-km radius of the outbreak sites. We selected 1 EHDV-8–positive blood sample from Sardinia for WGS; results confirmed that the Sardinia EHDV-8 strain (GenBank accession nos. OP897265–74) shares high nucleotide sequence identity (>99.9%) with multiple EHDV-8 TUN 2021–like strains. WGS of the Sicily strains is ongoing. We isolated the virus from all rRT-PCR–positive blood samples; all serum samples tested positive by ELISA and by virus neutralization (antibody titer 10–20).

Confirmation of novel *Orbivirus* incursion into the EU sustained by EHDV-8 was predictable, considering the distribution of this virus in Tunisia and likely in neighboring countries (*8*). On November 18, 2022, EHD was also reported in the Andalusia region of Spain, in the cities of Cadiz and Seville. Predicting future scenarios for the EU cattle production system is difficult, but EHD will probably pose new challenges to EU veterinary authorities. The lessons learned with bluetongue should be a reference for choosing proper control and prevention strategies for EHD. Overall, these events further emphasize the importance for countries in Europe to have robust collaborations with authorities in North Africa on public and animal health. The prompt detection of EHDV-8 in Sardinia and Sicily is the most recent example of the benefits that such relationships could yield. This collaboration proved crucial; it led to development of a specific and accurate molecular test for detecting EHDV-8, given that knowledge of the genome constellation and the genomic relatedness of EHDV-8 with extant EHDV serotypes had already been achieved. Vaccine development needs to be boosted because vaccination is the only strategy to reduce virus circulation and prevent direct and indirect economic losses.

## References

[1] Maclachlan NJ, Mayo CE, Daniels PW, Savini G, Zientara S, Gibbs EP. Bluetongue. Rev Sci Tech. 2015;34:329–40. 10.20506/rst.34.2.2360 26601438

[2] Savini G, Afonso A, Mellor P, Aradaib I, Yadin H, Sanaa M, et al. Epizootic heamorragic disease. Res Vet Sci. 2011;91:1–17. 10.1016/j.rvsc.2011.05.004 21665237

[3] Rushton J, Lyons N. Economic impact of Bluetongue: a review of the effects on production. Vet Ital. 2015;51:401–6. 26741252 10.12834/VetIt.646.3183.1

[4] Lorusso A, Guercio A, Purpari G, Cammà C, Calistri P, D’Alterio N, et al. Bluetongue virus serotype 3 in Western Sicily, November 2017. Vet Ital. 2017;53:273–5. 29307120 10.12834/VetIt.251.520.178

[5] Lorusso A, Sghaier S, Di Domenico M, Barbria ME, Zaccaria G, Megdich A, et al. Analysis of bluetongue serotype 3 spread in Tunisia and discovery of a novel strain related to the bluetongue virus isolated from a commercial sheep pox vaccine. Infect Genet Evol. 2018;59:63–71. 10.1016/j.meegid.2018.01.025 29386141

[6] Cappai S, Rolesu S, Loi F, Liciardi M, Leone A, Marcacci M, et al. Western Bluetongue virus serotype 3 in Sardinia, diagnosis and characterization. Transbound Emerg Dis. 2019;66:1426–31. 10.1111/tbed.13156 30806040 PMC6850434

[7] Calistri P, Giovannini A, Conte A, Nannini D, Santucci U, Patta C, et al. Bluetongue in Italy: Part I. Vet Ital. 2004;40:243–51. 20419672

[8] Sghaier S, Sailleau C, Marcacci M, Thabet S, Curini V, Ben Hassine T, et al. Epizootic haemorrhagic disease virus serotype 8 in Tunisia, 2021. Viruses. 2022;15:16. 10.3390/v15010016 36680057 PMC9866946

[9] Marcacci M, De Luca E, Zaccaria G, Di Tommaso M, Mangone I, Aste G, et al. Genome characterization of feline morbillivirus from Italy. J Virol Methods. 2016;234:160–3. 10.1016/j.jviromet.2016.05.002 27155238 PMC7172958

[10] World Organisation for Animal Health. Manual of diagnostic tests and vaccines for terrestrial animals 2022 [cited 2022 Nov 12]. https://www.woah.org/en/what-we-do/standards/codes-and-manuals/terrestrial-manual-online-access

